# Is the “*P*-value” Alone a Sufficient Metric for the Magnitude of Clinical Benefit and Which Clinical Trial Is Actually Positive, KEYNOTE-394 or KEYNOTE-240?

**DOI:** 10.1093/oncolo/oyad145

**Published:** 2023-06-09

**Authors:** Tulay Kus, Gokmen Aktas, Irfan Cicin

**Affiliations:** Department of Medical Oncology, School of Medicine, Gaziantep University, Gaziantep, Turkey; Gaziantep Medical Park Private Hospital, Gaziantep, Turkey; Department of Medical Oncology, School of Medicine, Trakya University, Edirne, Turkey

## Abstract

This commentary asks if, when determining the target *P* -value in a clinical study, it is sufficient to evaluate the study as “negative” or “positive” based on the target *P* -value alone, or whether this could lead to illusions.

Qin et al reported an important positive clinical study from Asia that demonstrated a survival advantage for pembrolizumab over placebo in patients with hepatocellular carcinoma (HCC) after first-line treatment.^1^ The primary endpoint of KEYNOTE-394 was overall survival (OS). Median OS was statistically longer in the pembrolizumab than the placebo arm (14.6 versus 13.0 months), with 2-year survival rates of 34.3% and 24.9%, respectively. The hazard ratio (HR) for death was 0.79 (95%CI: 0.63-0.99) with a *P*-value of .0180; below the prespecified one-sided significance threshold of .0193 for OS in the final analysis (Table 1). By comparison, KEYNOTE-240, a similarly designed phase III study, is referred to as a negative clinical trial despite its apparently better results. Overall survival for pembrolizumab and placebo was 13.9 and 10.6 months, respectively, with an HR (95%CI) of 0.781 (0.611-0.998). However, the *P*-value of .0238 failed to exceed .0174, the one-sided significance thresholds for the primary endpoints of OS and progression-free survival (PFS).^2^ Thus, despite achieving a better delta in median overall survival (3.3 versus 1.6 months) and similar lowest thresholds of HR (0.611 and 0.63) in KEYNOTE-240 compared to KEYNOTE-394, the latter was deemed a failure. In assigning magnitudes of clinical benefit, the ESMO guidelines consider the OS contributions of the experimental drug and HR values. For cancers with a survival expectancy of less than 12 months, the magnitude of clinical benefit of the experimental drug is highest if both the survival contribution is ≥3 months and the lowest HR boundary is ≤0.65 or its 2-year absolute survival contribution is ≥10%.^3^ Therefore, when looking to the ESMO Magnitude of Clinical Benefit Scales (MCBS) KEYNOTE-240 scores a “4” despite being considered a negative study, while the “positive” KEYNOTE-394, scores only a “2.”

**Table 1.** The results of 2 studies: KEYNOTE-394 and -240.

	KEYNOTE-394 (positive study)	KEYNOTE-240 (negative study)
Delta OS, months	1.6 [14.6–13.0]	3.3 [13.9–10.6]
OS, HR [95%CI]	0.79 [0.63-0.99]	0.781 [0.611-0.998]
*P*-value for OS	.0180	.0238
One-sided significance threshold	.0193	.0174

Abbreviations: HR, hazard ratio; OS, overall survival.

We thus ask if when determining the target “*P*-value” in a clinical study, even if the *P*-value corresponding to the expected survival contribution and HR of the drug is chosen, is it sufficient to evaluate the study as “negative” or “positive” based on the target *P*-value alone, or can this just lead to an illusion as to who actually won? Note here that if the *P*-value target for KEYNOTE-394 had been chosen as in KEYNOTE-240, the former would have actually been scored a negative study. Additionally, trivial differences often alter median OS values and, therefore, assessing clinical utility on the basis of median and delta OS values alone may be misleading, leading one study to appear to be more meaningful than the other. Finally, one could argue that despite a difference in the deltas of the median OS values, these 2 studies, with similar hazard ratios and *P*-values, actually have similar clinical implications. In KEYNOTE-394, for example, a better than expected median survival in the standard arm of 13.6 months compared to 10.6 months in KEYNOTE-240 could have contributed to the inferior delta OS of 1.6 months. Gradual improvement of the control arm with diminishing delta OS values may occur as the concept of “learning curve” comes into play with improvements in toxicity and patient management, as experience accrues with increased sequential use and experience with approved treatments. Thus, it may be reasonable to target the *P*-value at a weaker level in sequential study designs.^4^ However, compromising the target *P*-value can result in a “positive study” even as the magnitude of clinical benefit diminishes. Should we aim for a “positive study” or high level of clinical benefit? Is the “*P*-value” alone a sufficient target for the magnitude of clinical benefit? Which clinical trial is actually positive, KEYNOTE-394 or KEYNOTE-240?

In fact, these 2 studies are very similar and for the most part indistinguishable. Although both studies achieved *P*-values lower than .05, both can be considered negative, either because they did not reach the target *P*-value (KEYNOTE-240) or because they achieved the target but had low clinical utility (KEYNOTE-394). In some clinical studies, a *P*-value target <.05 is sufficient for statistical significance, while sometimes a target is set using a specific cutoff, as in these 2 studies. This latter target may vary for each cancer and drug type, but the specific *P*-value target chosen should provide meaningful clinical benefit. For example, as in these 2 studies evaluating a cancer with a proven second-line treatment, such as hepatocellular cancer, one might expect pembrolizumab to perform better than placebo and a *P*-value of .0238 may not be clinically significant despite being <.05. On the other hand, for a cancer type with limited treatment options, one may consider the results of these 2 studies significant. Should we allow a negligible difference in the cutoff *P*-value from .0174 to .0193 determine whether one drug is approved for use in clinical practice while another is thrown away? In this regard, in the study design, different goals should be set in the selection of clinical utility, or the evaluation of the study results should not be interpreted sharply as “target achieved” or “target not met” based on the *P*-value alone. These 2 studies, whose results are mostly close to each other, are actually a very good clinical example of how easily we can misinterpret the results of our pivotal clinical trials.
